# Integrating the ESM usage and work engagement for employee agility performance: based on regulatory focus theory

**DOI:** 10.1186/s40359-024-01833-3

**Published:** 2024-06-05

**Authors:** Yilinna Bao, Ye Zhu, Shamsa Kanwal, Ubaid Ullah

**Affiliations:** 1 School of Humanities, Jilin University, Changchun, 130012 Jilin Province China; 2 Academy of Psychology and Behavior, Tianjin Normal University, Tianjin Province, 300387 China; 3 School of Public Affair, University of Science and Technology, Hefei, China; 4 Employees’ Old Age Benefits Institution (EOBI), Islamabad, Pakistan

**Keywords:** ESM usage, Work engagement, Prevention focus, Promotion focus, Employee agility

## Abstract

This present research aims to clarify the intricate conjunction of enterprise social media (ESM) utilization and employee agility with a main focus on uncovering the underlying mechanisms that work through the mediating influence of work engagement and the moderating influence of regulatory focus. Drawing upon regularity focus theory, 353 Chinese samples of ESM users in organizational contexts are analyzed using SPSS 23.0. The empirical findings substantiate a robust and significant positive linkage between ESM usage and worker agility. Further reinforcing the model, the mediating role of work engagement is established as it channels the impact of ESM usage on worker agility. Turning to the moderating effects, the study unveils the differential impact of prevention focus and promotion focus, wherein individuals with a lower prevention focus exhibit a more pronounced positive linkage between ESM usage and worker agility. Similarly, individuals with a higher promotion focus demonstrate a heightened positive association between ESM usage and worker agility. By comprehensively inspecting the intricate dynamics of ESM usage, work engagement, and regulatory focus, this study enhances our theoretical understanding of how these factors synergistically shape employee agility, ultimately furnishing organizations with invaluable insights to foster and cultivate an agile workforce.

## Introduction

To leverage burgeoning opportunities within the dynamic marketplace, organizations are compelled to develop agile workforce that can respond to market competition [1, 2]. The potential benefits of employee agility have been extensively deliberated to enhance client service, product quality, and organizational knowledge acquisition [3–5]. However, the scarcity of studies exploring the means to foster such agility has become apparent. In line with, Cai, Huang [5] suggested that both enterprise social media (ESM) and psychological conditions may strengthen worker agility. On one hand, Hosein [6] proposed prevention focus and promotion focus as pivotal psychological factors that influence employee agility. The literature suggests that employee agility is based on frequent communication, information sharing, and collaboration with other employees.

On the other hand, the discussion of how ESM influences workers’ agility continues [7–10]. Mainly, ESM is perceived as advantageous in addressing unforeseen alterations, as it facilitates knowledge acquisition from colleagues [11]. On the contrary, other researchers note that ESM may be less proactive and reactive to market shifts because employees may misuse the online resource [12], leading to a higher degree of distraction [13], and an apparent feeling toward groupthink [11]. The polarity between these two arguments demonstrates the big room for additional investigation into the hidden processes that create the connection between ESM usage and agility outcome [14].

The prevalence of ESM offers valuable insights into the interplay between work engagement and worker agility [15]. ESM, a web-based application, facilitates intra-firm communication and message dissemination among individuals [7, 16]. It cultivates an interactive environment that fosters employee communication, knowledge sharing, and mutual comprehension [17, 18]. Through the aid of ESM, work engagement assumes a crucial role in enhancing a worker’s agility by providing valuable information for cognitive processing, and effectively aligning organizational members with their work roles [11]. The significance of employee engagement in establishing a truly agile workforce has been established by Arazy and Gellatly [19]. Muduli [20] research confirms the substantial contribution of employee involvement to workforce agility. Sumukadas and Sawhney [4] posit that employee engagement serves as a predictive factor for worker agility, noting that while low-level engagement practices possess the inherent potential to directly foster worker agility, it is the high-power practices that truly contribute to worker agility [21]. Active participation of individuals in all aspects of the organization allows for the infusion of their comprehensive ideas and energy, thereby ensuring survival and heightened productivity within the company [22].

Regulatory focus is a personality trait that refers to the way people approach goals [23]. Individuals with a promotion focus are encouraged by gains and rewards [24], while individuals with a prevention focus are motivated by avoiding losses and mistakes [25]. Research has shown that regulatory focus may moderate the connection between work ESM usage and worker agility [26]. For example, one research discovered that employees with a promotion focus are expected to benefit from work ESM usage than employees with a prevention focus [27]. This is because individual with a promotion focus are more likely to see ESM usage as a way to track their progress and receive feedback, which can help them to improve their performance. In comparison, individuals with a prevention focus are more likely to see work ESM as a way to be monitored and controlled, which can lead to anxiety and stress [28].

**Fig. 1:**
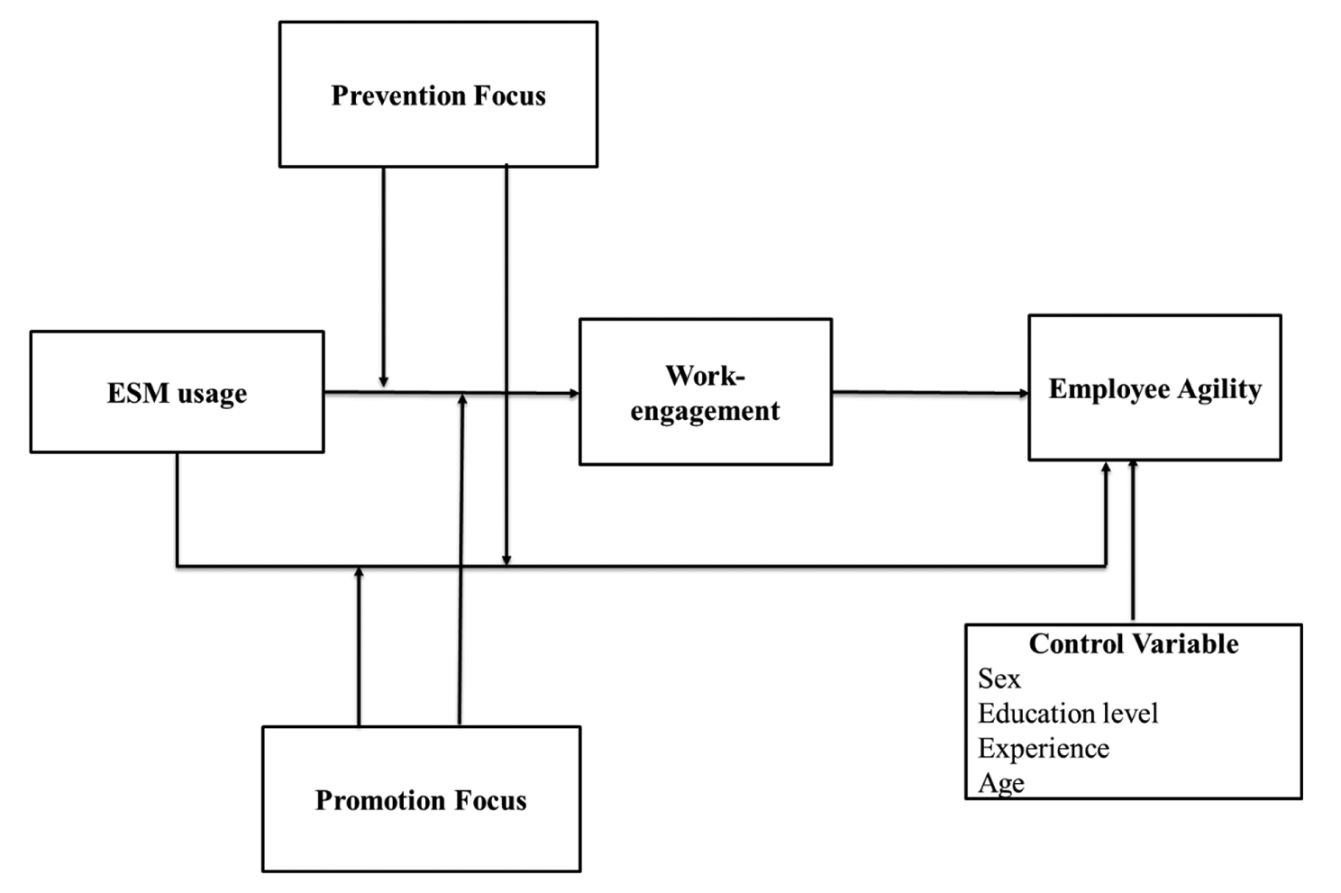
Conceptual Model

The present study aims to address the aforementioned issues by examining: (1) the linkage between ESM usage and worker agility, (2) the mediating influence of work engagement on this linkage, and (3) the moderating influence of prevention focus and promotion focus based on regulatory focus theory on the linkage between ESM usage and both work engagement and worker agility. This research endeavors to contribute to the present literature on ESM in three significant ways. Firstly, we delve into the emerging domain of enhancing employee agility by exploring the crucial yet contentious factor of ESM. By considering ESM usage as a catalyst for fostering individual agility, we advance this area of inquiry. Secondly, we investigate the impact of work engagement on the development and manifestation of employee agility, thereby shedding light on their interrelationship. Lastly, by examining the moderating roles of prevention focus and promotion focus in the linkage between ESM usage and both work engagement and employee agility, we offer insights into the effective utilization of a specific IT artifact to promote agility. The Fig. 1 indicates the overall conceptual model of the study.

## Theoretical background and literature review

### ESM usage

ESM, an innovative form of social media, has been specifically designed to facilitate the exchange of information, promote knowledge sharing among employees, encourage interactive employee engagement, and establish virtual communities within the organizational context [28–30]. It represents a burgeoning technological advancement that has gained traction among individuals in the workplace, as evidenced by notable studies [15, 31]. ESM is widely acknowledged as a potent instrument for fostering effective work-related communication and cultivating robust relationships among individuals [32–34].

ESM has gained widespread utilization for creating business value through fostering enhanced interaction, marketing initiatives, communication channels, and knowledge-sharing endeavors [35–37]. A multitude of companies have embraced ESM as a means to engage with consumers and forge strong connections with business partners [38]. Presently, an increasing number of companies in the e-commerce, manufacturing, and finance sectors have adopted ESM apps for various business purposes such as streamlined communication, efficient process direction, profitable marketing, society cultivation, secure data storage, and seamless online meetings [39]. ESM’s robust features encompass video/audio calls, instant messaging, reminder functionalities, comprehensive business reporting tools, seamless document exchange, and reliable web-based services [40]. Through ESM apps, members from diverse business units within an organization can consistently communicate and collaborate, irrespective of geographical barriers [41].

ESM serves as a powerful tool to bridge the gap created by power distance, facilitating the development and maintenance of professional relationships among a vast network of individuals [39]. It greatly enhances individual communication by enabling seamless collaboration and knowledge sharing among workmates, irrespective of geographic obstacles [42, 43]. Extensive literature highlights that ESM functions not merely as a distribution platform for information dissemination, but also as a conduit for disseminating information through the intricate web of social connections [44]. Scholars have observed various benefits linked with ESM, including socialization possibilities, increasing trust among individuals, successful marketing of goods, and information-sharing activities [45, 46]. Furthermore, research has confirmed that the utilization of ESM significantly increases information-sharing processes, facilitates continuous learning, and aids in informed decision-making [47]. Accordingly, within the realm of ESM, information is enriched with customer reviews and comments, which play a pivotal role in shaping purchasing decisions by providing valuable feedback [48, 49].

Notwithstanding the widespread recognition of the advantages of ESM [33, 50, 51], research examining its practical implementation in the workplace to enhance business outcomes is still in its nascent stages, primarily limited to conceptual inferences rather than concrete empirical evidence [26, 52]. Considering the prevalence and potential benefits associated with ESM, it is imperative for scholars to direct their attention toward comprehensively understanding its utilization within the organizational context.

The existing body of literature suggests that the utilization of ESM holds significant implications for employee performance [5]. Notably, ESM platforms offer innovative channels for knowledge exchange, development, dissemination, and facilitation of job-related educational opportunities for workers [8, 50, 53–56]. Through ESM, employees can swiftly exchange files, engage in text-based discussions, and resolve ambiguities, thus fostering the development of shared knowledge [54, 57, 58]. By sharing their experiences, employees can also benefit from the knowledge of others [44]. Previous research has demonstrated a positive connection between ESM usage and work outcomes [27]. Furthermore, ESM has been discovered to support connection formation and sustaining within the organizational setting, which is critical for the efficient sharing of information procedures [59, 60]. ESM technology enables workers to expand their social networks and cultivate trust among one another [18, 41].

### Employee agility

Agility encompasses the capacity and competence of an individual to swiftly react to abrupt shifts occurring within their organization’s internal and external environments [61, 62]. It holds sway over organizational performance and enhances survival prospects within the competitive business landscape [4, 63]. The advantages of employee agility are manifold, encompassing domains such as quality of goods, interaction with consumers, and acquiring knowledge [6, 63]. Worker agility can be organized into three distinct dimensions: adaptability, resilience, and proactivity [62, 64]. Adaptability pertains to employees’ adeptness in changing to alterations in the environment, resilience reflects a positive mindset towards change and the embracing of novel and unforeseen circumstances, while proactivity denotes employees’ proactive initiation of activities to resolve change-related issues and foster improvements in their work endeavors [2].

The scholarly literature highlights that the development of employee agility is closely linked to a flexible organizational structure that fosters a seamless flow of information and knowledge among employees [65, 66]. Cooperation and efficient interaction have emerged as critical aspects in improving employee agility since both promote the sharing of information among individuals [20, 22]. Moreover, researchers argue that access to relevant and timely information, as well as a clear understanding of the overall organizational direction, are vital for employees to effectively respond to changing environments [15]. Therefore, information assumes a central role in enabling employees to attain the agility required for navigating dynamic circumstances [67].

### Work engagement

Work engagement is described as a self-sustaining, persistent, pleasant, and fulfilling affective-cognitive and motivational-psychological state linked to work. This notion corresponds with the conceptual framework usually mentioned as the European Engagement Model, as supported by numerous research [19, 24, 68]. To operationalize this construct, the Utrecht Work Engagement Scale (UWES) encompasses three distinct subscales: vigor, dedication, and absorption. Vigor pertains to a worker’s levels of energy and mental resilience, their willingness to invest effort in their job, and their ability to persist in the face of challenges [69]. Dedication reflects an individual’s emotional involvement and identification with their job, supported by sentiments, encouragement, inspiration, pride, and an awareness of achievement. Absorption captures an employee’s state of complete immersion and intense concentration in their work, often leading to a loss of track of time and difficulty in disengaging from their tasks. An engaged employee demonstrates enthusiasm, exerts high levels of energy in their job, and experiences difficulty detaching from their work [68].

Work engagement has received an extensive amount of attention and empirical evidence as a critical component of employee engagement in recent scholarly research. However, it is not immune to critique. According to prior research, work engagement is related to recognized variables like job participation, job satisfaction, and organizational commitment [9, 24]. In contrast, other research emphasizes the distinctness of work engagement when juxtaposed with other worker behaviors [21, 70–72].

### Prevention focus and promotion focus

Higgins (1997, 1998) introduced the regulatory focus theory (RFT), which combines the desire for pleasure and the prevention of distress with self-regulation of emotions, behaviors, and cognitive processes. This theory establishes two distinct self-regulatory systems, namely the promotion regulatory focus and the prevention regulatory focus, which operate independently and guide individuals’ strategic choices in approaching desired outcomes and avoiding undesirable ones. It is essential to consider that regulatory focus is orthogonal to the traditional approach/avoidance motivation framework, as it encompasses both the pursuit of desired outcomes and the avoidance of undesired outcomes. These facets of regulatory focus give rise to unique goal preferences, intentions, and motivations. The antecedents of regulatory focus include individual needs, values, and the framing of situational contexts [23]. While regulatory focus is generally considered a stable trait, it can be influenced by changes in the environment or personal circumstances, leading to shifts in an individual’s regulatory focus state [73]. Individuals oriented towards promotion tend to prioritize positive outcomes such as profit or gains, whereas those with a prevention focus prioritize stability and guarding against potential negative consequences such as harm or loss. These two orientations lead to distinct motivational states and are logically independent of each other, meaning it is not feasible to possess high levels of both, only one, or neither.

These two regulatory techniques, promotion, and prevention, are anchored on fundamental endurance demands like nourishment and protection [23, 74, 75]. Employees with a promotion focus are primarily driven by the pursuit of positive outcomes or gains, while those with a prevention focus are encouraged by the need to ensure security, fulfill commitments, and meet obligations [24, 75, 76]. Promotion-focused individuals are oriented toward achieving desirable results, while prevention-focused individuals are more concerned with avoiding undesirable outcomes [77, 78]. It is essential to understand that although promotion and prevention focus are interconnected, they have distinct motivational states [75]. Empirical research has revealed the symmetry of promotion and prevention foci, suggesting that these regulatory states can be independently examined [79–81].

In this study we propose that the regulatory foci of promotion and prevention play a significant function in moderating the connection between ESM usage and both worker agility and work engagement, thereby influencing the ultimate level of employee agility within organizations. Research has shown that individuals with a higher promotion focus exhibit a stronger significant connection between ESM usage and employee agility [19, 27]. This suggests that a motivational orientation focused on growth, achievement, and advancement enhances ESM utilization’s impact on fostering employee agility. Conversely, individuals with a higher prevention focus demonstrate a weaker association between ESM usage and employee agility [82]. This regulatory focus, characterized by an emphasis on security and risk aversion, may inhibit the exploration and utilization of ESM platforms, limiting their potential to enhance employee agility. Additionally, the moderation impact of promotion and prevention focus extends to the connection between ESM usage and work engagement, which in turn influences employee agility.

### Hypothesis development

#### ESM usage and employee agility

The use of ESM has become increasingly prevalent in organizations, providing employees with a platform for communication, cooperation, and information sharing. Previous research suggests that ESM can positively impact employee agility, which indicates the capacity of an employee to respond and adapt to changing circumstances in a dynamic work environment [2, 5]. ESM platforms offer employees the opportunity to access real-time information, connect with colleagues across departments and hierarchical levels, and engage in rapid decision-making processes. Such features facilitate the exchange of knowledge, enhance communication channels, and promote cross-functional collaboration. By leveraging these capabilities, employees are better equipped to stay informed, make timely decisions, and adapt their work practices to changing organizational demands, ultimately enhancing their agility within the organization [44]. Therefore, it is important to investigate the connection between ESM usage and worker agility to gain a deeper knowledge of how these technologies can contribute to organizational effectiveness.

Existing literature provides empirical evidence supporting the connection between ESM usage and worker agility. For instance, a study conducted by Kang, Jiang [44] investigated the impact of ESM on organizational agility in a large multinational company. The results demonstrated that increased usage of ESM tools positively influenced employee agility, leading to enhanced responsiveness to market changes, improved decision-making, and increased organizational innovation. Similarly, Pitafi, Kanwal [83] examined the role of ESM adoption in fostering employee agility in a sample of technology firms. The findings revealed a positive connection between ESM usage and employee agility, with employees reporting higher levels of adaptability, learning orientation, and activeness when utilizing ESM platforms. These studies offer empirical evidence for the assumption that ESM adoption is positively associated with worker agility, emphasizing ESM technologies’ ability to improve personal level outcomes throughout organizations. Along with the theoretical underpinning and real-world proof, we suggest the following hypothesis:

##### H1

*There is a positive connection between ESM usage and employee agility, such that increased utilization of ESM platforms will be associated with higher levels of employee agility within the organization.*

#### Mediating role of work engagement on ESM usage and employee agility

Work engagement has been acknowledged as a crucial factor in determining employee performance and organizational outcomes [21, 84]. It refers to an attractive, satisfying, and robust state of mind distinguished by vigor, devotion, and concentration [84]. In recent years, the use of ESM has gained significant consideration as a tool for enhancing organizational communication and collaboration [39]. ESM platforms enable employees to connect, share knowledge, and collaborate across organizational boundaries, thereby facilitating information exchange and problem-solving [85]. Given the potential benefits of ESM, it is important to understand how it influences employees’ psychological states and behaviors. Therefore, investigating the mediating role of work engagement in the linkage between ESM usage and worker agility becomes imperative.

Employee agility, defined as the capability to rapidly adapt, learn, and innovate in reaction to changing work demands [61], has gained prominence due to the dynamic and uncertain nature of the contemporary business environment. It allows organizations to react effectively to competitive changes and obtain new possibilities [20]. Work engagement has been identified as a potential mechanism that influences employees’ agility levels [5, 67]. Engaged employees exhibit higher levels of proactive behavior, initiative, and flexibility, which are vital components of employee agility [9]. Therefore, it is plausible to suggest that work engagement may mediate the connection between ESM usage and worker agility, as engaged employees are likely to be more receptive to the opportunities and knowledge exchange facilitated by ESM platforms, leading to increased agility in their work behaviors. Building on the above theoretical background, we propose the following hypothesis:

##### H2

*Work engagement mediates the relationship between ESM usage and employee agility, such that higher levels of ESM usage will be positively associated with increased work engagement, which in turn will be positively associated with higher levels of employee agility.*

#### Moderating role of prevention focus

Recent literature suggests that the moderating role of Prevention focus may significantly influence the linkage between ESM usage and worker agility. Building upon Higgins [23] theoretical framework on prevention focus, several studies have proposed that employees with a higher Prevention focus tend to exhibit more cautious behaviors and are motivated to prevent the occurrence of potential negative outcomes [26]. Consequently, employees oriented towards prevention may demonstrate a more cautious demeanor in the workplace, given their heightened concern for task outcomes. On a public platform like ESM, which inherently exposes information, prevention-focused individuals may experience feelings of threat due to their inclination to safeguard their knowledge or information [86]. Following this notion, it is proposed that employees with a greater Prevention focus will moderate the linkage between ESM usage and employee agility. Specifically, it is expected that the positive linkage between ESM usage and worker agility will be stronger for individuals with a lower Prevention focus, as they may be more open to exploring novel ideas and engaging in proactive behaviors (Baranik et al., 2018). Conversely, individuals with a higher Prevention focus may demonstrate a weaker association between ESM usage and worker agility due to their tendency to prioritize stability and risk avoidance (Gong et al., 2019). Therefore, it is crucial to investigate the role of Prevention focus as a potential moderator to enhance our knowledge of the linkage between ESM usage and employee agility. Therefore, we hypothesize that:

##### H3

*The linkage between ESM usage and employee agility is moderated by an individual’s prevention focus, such that the employees with a lower prevention focus will exhibit a stronger positive connection between ESM usage and employee agility, whereas employees with a higher prevention focus will demonstrate a weaker association between ESM usage and employee agility.*

Further, we propose that prevention focus, as an individual difference variable, moderates the connection between ESM usage and work engagement. Prior studies have indicated that employees with a prevention focus tend to maintain their current positions, fulfill their duties and tasks within the organization, and potentially avoid unexpected circumstances [19, 82]. Promotion-focused individuals with a promotion focus may view information sharing as conducive to personal growth and achievement [26]. In the context of ESM’s open and public nature, prevention-oriented employees may perceive it as a risk to their security and safety, leading them to limit the exchange of work-related information [19]. Building upon previous research suggesting that prevention-focused individuals tend to prioritize security, avoid losses, and maintain stability (Higgins, 1997), we anticipate that prevention focus will attenuate the significant connection between ESM usage and work engagement. Specifically, individuals characterized by pronounced prevention focus might perceive ESM as encroaching upon their autonomy and potentially disruptive to their established routines, leading to a weaker affiliation between ESM usage and work engagement. Conversely, individuals with a lower prevention focus might view ESM as a facilitator of communication and collaboration, thus fostering a stronger connection between ESM usage and work engagement. Thus, we predict that:

##### H4

*Prevention focus moderates the positive relationship between ESM usage and work engagement, such that the connection is weaker for individuals with a high prevention focus compared to those with a low prevention focus.*

#### Moderating role of promotion focus

As an individual difference variable, promotion focus moderates the connection between ESM usage and employee agility. Drawing from previous research suggesting that promotion-focused individuals tend to seek growth, approach gains, and exhibit a desire for advancement (Higgins, 1997), we anticipate that promotion focus will strengthen the positive linkage between ESM usage and worker agility. When individuals exhibit a high degree of promotion focus, they become particularly attuned to significant outcomes [87]. Employees may perceive that sharing information through ESM aids in enhancing their resources, trust, and esteem [26, 45]. Moreover, those with a promotion focus may offer support and assistance to colleagues in resolving work-related issues, as such actions elevate their visibility and standing within the workplace [26]. Specifically, individuals with a higher promotion focus may observe ESM as a valuable tool for acquiring feedback, monitoring their progress, and adapting to changing work demands, thereby enhancing their agility in the workplace. In contrast, individuals with a low promotion focus may view ESM as less relevant or may be less motivated to utilize its potential benefits, resulting in a weaker linkage between ESM usage and worker agility. Thus, we forecast that promotion focus will moderate the positive linkage between ESM usage and worker agility, with higher levels of promotion focus strengthening this association. Hence we hypothesis here that:

##### H5

*Promotion focus moderates the positive linkage between ESM usage and employee agility, such that the connection is stronger for individuals with a high promotion focus compared to those with a low promotion focus.*

Additionally, drawing on the motivational framework of promotion focus, employees with a high promotion focus are likely to perceive ESM as a valuable tool for networking, showcasing their accomplishments, and accessing career opportunities. Consequently, we anticipate that these individuals will experience higher levels of work engagement when utilizing ESM. Conversely, individuals with a low promotion focus may view ESM as less relevant to their career advancement or may exhibit lower motivation to actively engage with it. As a result, we expect a weaker connection between ESM usage and work engagement for individuals with a low promotion focus.

##### H6

*Promotion focus moderates the positive linkage between ESM usage and work engagement, such that the connection is stronger for individuals with a high promotion focus compared to those with a low promotion focus.*

## Research method

### Research instruments

All the survey items employed in this research were taken from the scales that have been validated by prior studies. The survey items were rated on a “5-point Likert Scale” ranging from “1 = Strongly Disagree” to “5 = Strongly Agree”. Further considering the nature of Chinese respondents [88] the initial English items were converted into Chinese language by translation software. For proofreading and accuracy of all the items, we invited three Chinese experts to correct the wording and translation mistakes. While reviewing the initial questionnaire with the translated variant, no major variation was noticed. As a result, the Chinese version was used for final data collection. The construct of worker agility is measured using three dimensions that include proactivity, adaptability, and resilience and is adapted from Alavi, Abd. Wahab [89]. The instruments of proactivity, adaptability, and resilience are measured using 8, 5, and 6 items respectively. The measurement item of work engagement consists of 5 items and is adapted from Sun, Wu [84]. The construct ESM usage includes five items and is adapted from Cai, Huang [5]. The measurement items of promotion focus are measured using the items of Chen, Wei [26]. The instrument of prevention focus is measured using seven items of Chen, Wei [26]. Given that demographic characteristics might impact employee behavior [90], we controlled respondents’ gender, age, educational level, tenure, and position.

### Data collection methods

To analyze the proposed research model, the research relied on a survey design to get responses from Chinese employees in different firms. The reason for choosing China as a place of study was due to multiple factors. First, ESM is extensively used by Chinese employees for work-related communication because the Chinese government only allows access to selected public social media platforms and most public social media application is banned in the country. Chinese organizations widely employ ESM tools for their employee’s work-related communication. Second, China is considered a developed nation because of its economic strength and technological advancements [5]. Thirdly, most of the studies related to employee agility performance have been conducted in Western countries, Chinese employees have their own culture and are based on collaboration work. In addition, the author has also collaborated with Chinese educational institutions to validate the reliability of survey items and to acquire valid and accurate responses. This institution is widely recognized for its employee training programs, especially those focusing on information technology. With the help of the management of the institute, the authors have easily contacted the companies that allowed employees to use ESM for work-related communication. Companies’ managers were also informed that their feedback was only used for academic purposes and was kept confidential. Before conducting a large-scale survey author also conducted a pilot study on sixty-two respondents and the result was found satisfactory. The results of the pilot study indicated that Cronbach’s alpha (CA), and composite reliability (CR) values are higher than the proposed value of 0.70 [91].

In March 2023, we distributed 450 survey questionnaires to employees who adopted ESM technology. After that, we followed the respondents using phone calls and sent reminder messages to increase the response rate. Additionally, the collaboration behavior of higher management efficiently improved our response rate. During the discussions with managers, it was discovered that their primary goal of using ESM in organizations was to increase employee interaction, socialization, and information sharing. In response, we obtained 400 surveys over four weeks and deleted 47 inaccurate questions. Finally, we received 353 proper responses (a response rate of around 77.48%). Among the participants, 61.8% are male and 38.2% are female. More than half of the respondents are aged between 21 and 40 years. The majority of respondents obtained a graduate degree (51.0%), and 32.0% had a job tenure between 4 and 5 years. Further, we followed the methodology of Armstrong and Overton [92] and evaluated the possibility of non-response bias. By comparing the chi-squares of the key variables from the first 25% of the participants to those from the last 25%, we observed no statistically significant distinction between these two sets of measurement items. This finding revealed that non-response bias was not a serious issue in this study. The detailed summary of all the participants is illustrated in Table 1.

**Table 1:** Demographics

Variables	*N*	Percentage	Variables	*N*	Percentage
**Gender**			**Qualification**		
Male	218	61.8	Under-graduate	66	18.7
Female	135	38.2	Graduate	180	51.0
**Age**			Masters or above	107	30.3
Between 21–30	87	24.6	**Experience**		
Between 31–40	102	28.9	Less than- 1 year	37	10.5
Between 41–50	122	34.6	2–3 years	101	28.6
> 50 year old	42	11.90	4–5 years	113	32.0
			More than 5 years	102	28.9

## Data analysis and results

### Measurement model

The measurement model of the research was evaluated using the validity and reliability analysis of all the constructs. Specifically, a validity test may be obtainable by convergent validity and discriminant validity, whereas a reliability test can be performed by Cronbach’s alpha (CA), composite reliability (CR), and average variance extracted (AVE). The proposed theoretical framework is adequate when CA values are higher than 0.70 [93]. The conclusion of Table 2 indicated that all the constructs have CA values ranging from (0.87 to 0.96), higher than the proposed value of 0.70 [91]. The findings also indicated that all the values of CR range from 0.90 to 0.96 superior to the mentioned value of 0.70 [94]. Similarly, Table 2 results also reflected that all the constructs have AVE values ranging from (0.60 to 0.81) better than the suggested value of 0.50 [95]. All these results confirmed that all the constructs used in the research model have the appropriate level of convergent validity.

**Table 2:** Results of measurement analysis

Constructs	Items	Loadings	Cronbach α	Composite Reliability	AVE
Promotion focused	8	0.68–0.92	0.96	0.87	0.77
Prevention focused	7	0.79–0.95	0.95	0.81	0.81
Proactivity	8	0.78–0.89	0.94	0.95	0.71
Adaptability	6	0.65–0.89	0.87	0.90	0.60
Resilience	5	0.79–0.88	0.92	0.94	0.77
ESM usage	6	0.61–0.85	0.93	0.85	0.71
Work engagement	5	0.74–0.94	0.92	0.93	0.73

Note: AVE = average variance extracted

Furthermore, we also observed the discriminant validity of the proposed research model using different approaches. First, we noticed the pair-wise correlation values of all the factors and AVE square roots of all the variables. The findings of Table 3 indicated that all the AVE square root values are higher than the inter-correlation values of all the constructs [93]. Moreover, the high correlation between constructs is 0.57 which is lesser than the standard value of 0.70. Second, we also computed the item loading and cross-loading of all the items as shown in Table 3. Findings of Table 4 indicated that all constructs were well-loaded in their respective columns and poorly loaded in other columns [5]. As a consequence, all the observations and results indicated that the proposed constructs possess an adequate level of discriminant validity.

**Table 3:** Correlation matrix and mean, standard division

Construct	Mean	SD	1	2	3	4	5	6	7	8	9	10	11
1. Promotion focused	3.64	1.04	**0.87**										
2. Prevention focused	3.79	0.80	0.18**	**0.90**									
3. Proactivity	3.97	0.76	0.10	0.09	**0.84**								
4. Adaptability	4.00	0.61	0.29**	0.40**	− 0.018	**0.77**							
5. Resilience	3.59	0.76	0.06	0.10	-0.16**	0.15**	**0.87**						
6. ESM usage	3.66	0.83	0.63**	0.30**	0.06	0.28**	0.14**	**0.84**					
7. Work engagement	3.61	0.77	0.44**	0.38**	0.01	0.27**	0.22**	0.41**	**0.85**				
8- Experience	**NA**	**NA**	-0.21**	0.07	-0.12*	0.09	-0.04	-0.17*	-0.04	**NA**			
9- Education	**NA**	**NA**	0.11*	0.05	0.01	-0.08	-0.01	0.11*	0.17**	-0.31**	**NA**		
10- Age	**NA**	**NA**	-0.26**	-0.13*	0.02	0.03	0.12	-0.11*	-0.17**	0.17**	-0.34**	**NA**	
11- Gender	**NA**	**NA**	-0.03	-0.10	0.05	-0.05	-0.07	-0.03	0.01	-0.18**	0.11*	0.06	**NA**

Note: *p < 0.05, **p < 0.01

**Table 4:** Results of hypothesis testing

	B	SE	t	*R* ^2^
**Outcome: Work engagement**				**0.34**
Constant:	-0.03	0.04	-0.75	
ESM usage	0.27	0.06	4.31**	
Prevention focused	0.22	0.05	4.32**	
Promotion focused	0.29	0.06	4.95**	
ESM usage * Prevention-focused	-0.18	0.05	-3.65**	
ESM usage * Promotion focused	0.16	0.04	3.87**	
Experience	0.05	0.04	1.31	
Education	0.06	0.05	1.39	
Age	-0.05	0.04	-1.04	
Gender	0.03	0.04	0.82	
**Outcome: Employee agility**				**0.20**
Constant:	-0.03	0.03	-1.06	
ESM usage	0.07	0.04	1.99*	
Work engagement	0.03	0.03	0.83	
Prevention focused	0.13	0.03	3.97**	
Promotion focused	0.10	0.04	2.73*	
ESM usage * Prevention focused	-0.07	0.03	-2.34*	
ESM usage * Promotion focused	0.07	0.03	2.75*	
Experience	-0.04	0.02	-1.63	
Education level	-0.03	0.02	-1.23	
Age	0.07	0.02	3.01	
Gender	0.01	0.02	0.05	
Indirect effect of ESM usage on employee agility through work engagement	**Effect**	**SE**	**LLCI**	**ULCI**
	0.04	0.01	0.02	0.06

Note: *p < 0.05, **p < 0.01

### Common method variance (CMV)

We observed the guidelines of previous studies [96] and performed the CMV analysis employing multiple approaches. First, the questionnaire was designed to minimize CMV at the participant phase [46]. For this objective, one backward question was used to catch the attention of respondents while they answered the questions. Next, Herman’s single-factor approach was employed to analyze the possibility of CMV in the data set. This approach did not identify the presence of CMB in the data set since a single component only explained 25.07% of the entire variance, which was considerably lower than a maximum threshold of 50% [97]. Furthermore, variance inflation factor (VIF) assessment was employed to observe the possibility of CMB. The analytical findings showed that VIF values were lower than the minimal value of 3.30 [98], confirming that CMB was not a basic problem in the current study. As a consequence, all of the findings indicated that the CMB is not a significant concern for this research.

### Hypothesis testing

We conducted the analysis and generated the results of this study by following the approach of previous studies [99]. Model 10 of PROCESS Macro for SPSS was used as shown in Table 4. The outcome of Table 4 specified that ESM usage has a positive effect on employee agility (β = 0.07, *p* < 0.05), thereby H1 is confirmed by the current study. Further, we also hypothesized that work engagement mediates the linkage between ESM and worker agility. For mediation analysis, we employed the bootstrapping sampling technique on 5000 samples [100]; the findings produced a bootstrap of 95% confidence intervals (CIs) to acquire an indirect effect of ESM usage on worker agility through work engagement. The findings of Table 4 also confirmed that work engagement mediates the linkage between ESM usage and worker agility because (LLCI = 0.02 and ULCI = 0.06) not include zero, hence H2 is validated by existing study.

The moderation function of prevention-focus and promotion-focus is also analyzed and reported in Table 4. The outcome of Table 4 specified that Prevention focus lessens the relation between ESM usage and work engagement because the interaction term (β = -0.18, *p* < 0.01) specifies a significant relationship, H3 is also authenticated by this data. Similarly, outcomes of Table 4 specified that prevention focus reduces the link between ESM usage and employee agility (β = -0.07, *p* < 0.05), thereby supporting H4. In contrast, promotion focused on strengthening the association of ESM usage and work engagement with interaction terms (β = 0.16, *p* < 0.01), thereby supporting H5. Promotion focus also strengthens the association between ESM usage and worker agility (β = 0.07, *p* < 0.01), H6 is also authenticated by this data.

**Fig. 2:**
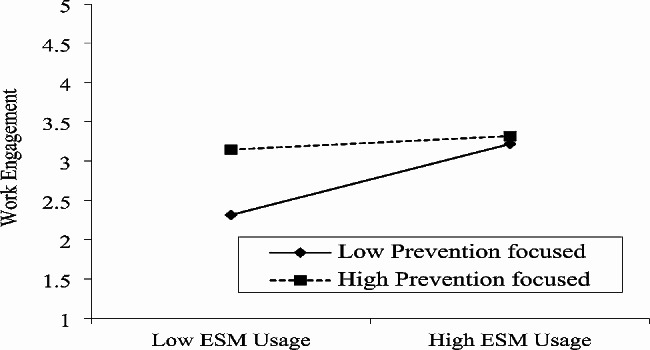
Moderating effect of prevention focus in the relationship between ESM usage and work engagement

**Fig. 3:**
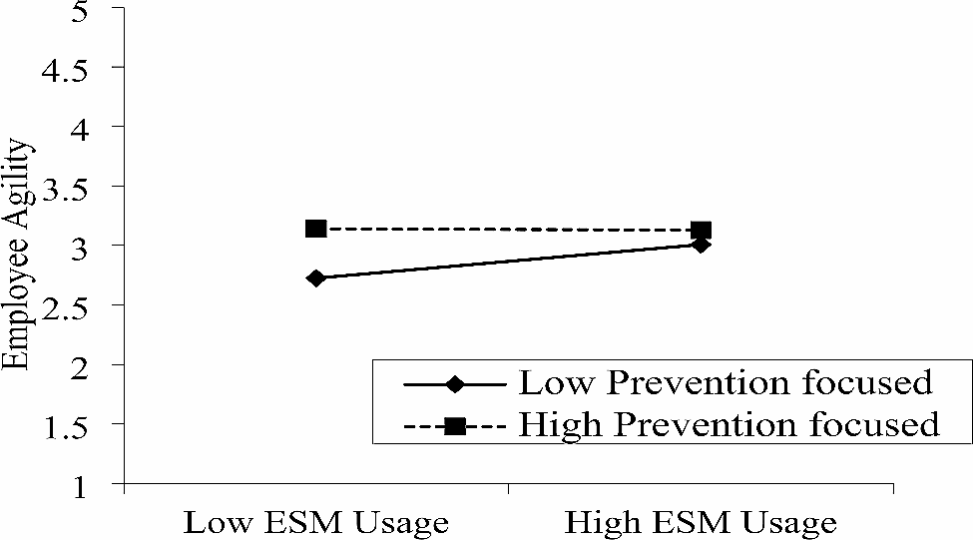
Moderating effect of prevention focus in the relationship between ESM usage and employee agility

**Fig. 4:**
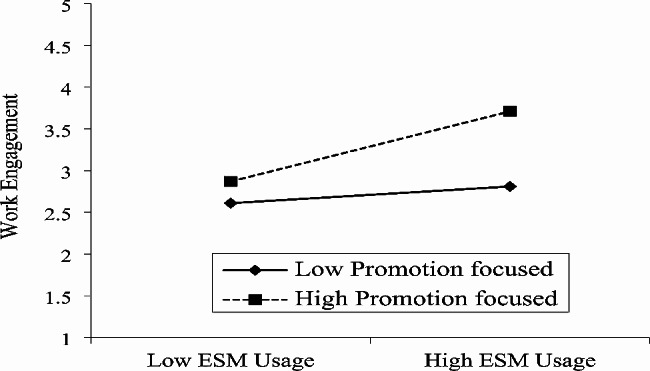
Moderating effect of promotion focus in the relationship between ESM usage and work engagement

**Fig. 5:**
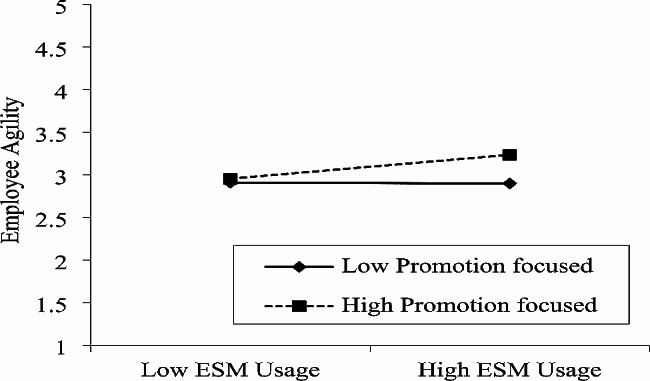
Moderating effect of promotion focus in the relationship between ESM usage and employee agility

Finally, we also used a graphical approach and further clarified the moderating role of prevention focus and promotion focus [101]. Fig. 2 indicates that when the prevention focus is higher connection between ESM usage and work engagement is lower. Similarly, Fig. 3 indicates that at a higher level of prevention-focused the relation between ESM usage and worker agility is lower. In contrast, Fig. 4 revealed that when the promotion focus is higher the connection between ESM usage and work engagement is also higher. Finally, Fig. 5 indicates that at a higher level of promotion focused the linkage between ESM usage and worker agility is also higher.

## Discussion, limitations, and implications

### Discussion

The present investigation aimed to examine the function of ESM usage concerning employee agility, focusing on the mediating role of work engagement and the moderating effects of individuals’ regulatory focus. Based on the literature emphasizing the importance of efficient information processing for employee agility [102, 103], we proposed that ESM usage facilitates the processing of valuable information, thereby enhancing employee agility. Furthermore, we predicted that work engagement mediates the connection between ESM usage and worker agility. The empirical investigation in this study validated the proposed positive connection between ESM usage and worker agility. These findings align with previous research highlighting the positive effects of utilizing modern technology on individual performance [102]. The results indicate that ESM usage has a significant role in enhancing employee agility within organizations.

The findings reveal a comprehensive and robust association between the utilization of ESM platform utilization and employee agility, affirming a positive relationship between these variables. Consistent with prior research examining the correlation between technology adoption and individual performance [102], the present result offers an indication to support the notion that heightened ESM usage is linked to elevated levels of employee agility within the organizational context. The outcomes underscore ESM’s role in empowering employees to efficiently process and leverage valuable information, enabling them to promptly adapt and respond to dynamic external changes.

Furthermore, the investigation revealed a nonlinear connection between ESM usage and employee agility, characterized by a distinctive U-shaped curve. This nonlinearity suggests that, as ESM usage intensifies, employee agility initially experiences a decline, only to surge after surpassing a specific threshold of ESM utilization. This intriguing pattern sheds light on the intricate dynamics that govern ESM usage’s influence on employee agility. The plausible reasoning behind this trend lies in the potential initial overwhelming employees may face when confronted with a flood of information as a result of heightened ESM usage. This initial decrease in agility is mitigated as employees become more adept at harnessing ESM platforms, gaining proficiency in information navigation, and leveraging new connections and relationships. These developments enable employees to effectively acquire, process, and utilize information, thereby bolstering their agility in navigating evolving work environments.

Crucially, this study also demonstrates that work engagement acts as a mediator construct in the connection in the link between ESM usage and worker agility. The analysis revealed that work engagement positively influences employee agility, serving as a conduit through which the effects of ESM usage manifest. These results suggest that ESM’s beneficial impact on employee agility is channeled through the enhancement of work engagement. By fostering a sense of dedication, involvement, and absorption among employees through ESM adoption, organizations create an environment conducive to agile behaviors, encouraging individuals to react efficiently to external changes and navigate dynamic work demands.

Regarding prevention focus, the results demonstrate that employees with a lower prevention focus exhibit a stronger positive connection between ESM usage and worker agility. This suggests that individuals who are less focused on avoiding negative outcomes and are more open to taking risks are more likely to benefit from ESM usage in terms of enhancing their agility. These individuals may be more inclined to explore and utilize ESM platforms’ features and functionalities, enabling them to efficiently process information, adapt to changes, and respond swiftly to emerging challenges. Similarly, the findings reveal the moderating role of promotion focus on the association between ESM usage and worker agility. Employees with a higher promotion focus demonstrate a stronger association between ESM usage and employee agility. This suggests that individuals who are driven by aspirations for growth, achievement, and advancement are more likely to harness the benefits of ESM platforms to enhance their agility. Promotion-focused individuals may be motivated by ESM’s opportunities for networking, knowledge sharing, and skill development, enabling them to be more agile in responding to changes and seizing new opportunities.

### Limitations and future research

Considering the overall idea and implementation of this research, it is essential to critically assess its contributions, while recognizing its limitations. Firstly, it is essential to recognize that factors beyond those explored in this study may potentially exert an influence on employee agility. For instance, researchers could expand the boundaries of this investigation by investigating how work factors such as job responsibilities, autonomy at work, and encouragement from managers affect employee agility [67].

Secondly, it is worth noting that the main variables of the present investigation were examined through subjective perceptions reported by distinct respondents. This inherently subjective nature of measurement introduces the possibility of common method bias. Although our analysis did not indicate significant concerns regarding this bias, future researchers should employ objective data or utilize multiple data sources to enhance the measurement accuracy. Additionally, the subjective nature of the measurement contributes to the high correlation observed between work engagement and employee agility. Though our examination suggests that multicollinearity was not a substantial concern in our dataset, there remains a need for improved methods to measure these two constructs. Furthermore, a larger sample size would enhance the statistical power and robustness of this dataset.

Thirdly, it is essential to recognize that the present study adopted a unidimensional concept of work engagement, despite the understanding that the impact of work engagement is largely contingent on other factors within the work environment. Consequently, we recommend that scholars further investigate the effects of work engagement by considering additional work environment factors. Furthermore, as ESM is mainly employed to encourage conversation among teammates, this study may be expanded to a multilevel analysis to evaluate the impact of ESM use in group-level contexts.

### Theoretical implications

The finding supports the current body of knowledge on the role of individual agility by making three significant theoretical advancements. Firstly, it addresses an important yet often overlooked topic, which is how to cultivate worker agility to appropriately respond to sudden changes [15]. The assessment of the factors of worker agility aligns with the call made by Alavi and Wahab [10] for further empirical studies in this area. Specifically, the theory of information processing is applied to effectively frame the concept of employee agility in terms of well-managed information [3]. Importantly, prior studies on how ESM usage affects employees’ outcomes often draw from social network theory, social capital theory, knowledge transfer theory, and meta-knowledge theory, but studies rarely explore the role of regularity focus theory. By adopting ESM platforms to handle a diverse range of information efficiently, employees can obtain the essential information to swiftly sense and respond to sudden changes. The outcome emphasizes the crucial role of information in fostering agility and urges scholars to explore ways to enhance workers’ information processing capacities by utilizing digital technology to achieve agility.

Secondly, the present study expands our knowledge of the deeper processes through which ESM usage influences worker agility. By establishing work engagement as a significant mediator in this relationship, this study highlights the importance of employees’ psychological states in harnessing the benefits of ESM platforms for agility. This finding extends the studies by emphasizing the role of employees’ active and emotional connections with their work as key drivers of agility. By investigating the mediating mechanism of work engagement in the connection between ESM usage and worker agility, the present study responds to the call from Cai, Huang [5] to deepen understanding of the link between ESM usage and worker agility through the lens of work engagement, providing detailed insights into how ESM usage influences employee agility.

Thirdly, the identification of prevention focus and promotion focus as significant moderating factors enhances our understanding of how individual regulatory orientations influence the connection between ESM usage and worker agility. This finding focuses on the importance of considering employees’ regulatory focus when implementing ESM initiatives to promote agility. Organizations can tailor their strategies to align with employees’ regulatory orientations, thus maximizing ESM platforms’ effectiveness in facilitating agility. Further, the study extends theoretical knowledge by shedding light on the role of prevention focus and promotion focus as moderators between ESM usage and work engagement. By recognizing the differential effects of these regulatory orientations, organizations can leverage ESM platforms to enhance work engagement in a targeted manner. Individuals with a high prevention focus may benefit more from ESM usage in terms of work engagement, whereas those with a high promotion focus may exhibit a stronger positive connection between ESM usage and work engagement. These findings underscore the need to consider individual differences in regulatory focus when designing interventions to promote work engagement through ESM platforms.

### Managerial implications

The outcome of the present study may assist managers in deciding how to use work engagement and ESM to increase worker agility. The use of ESM platforms among workers should first be actively encouraged and facilitated by organizations. Employees can acquire timely information efficiently through ESM, which improves their agility. Businesses may foster a culture of cooperation and information-sharing that promotes employee agility by encouraging the adoption and use of ESM platforms [35]. For instance, managers may encourage employees to utilize ESM for tracking requests from colleagues and providing assistance, or for acquiring knowledge from others within the organization. They could also promote ESM usage for monitoring task progress, improving task management, collaborating with colleagues, and accessing information resources. The use of ESM can enhance employee initiative, facilitate resource acquisition, and bolster agility to effectively navigate environmental changes.

Second, managers should prioritize initiatives that foster work engagement among employees. The present research highlights the mediating role of work engagement in the connection between ESM use and worker agility. Creating a work environment that promotes enthusiasm, dedication, and absorption can enhance employee engagement with their work and enable them to fully utilize ESM platforms for agility-related activities [84]. Organizations can achieve this by providing meaningful work, fostering positive relationships, and offering opportunities for skill development and growth. Managers are suggested to design some tasks on the ESM platform and discuss task-related issues on ESM to motivate the employees to use ESM for work-related activities.

Third, managers need to recognize and address individual differences, such as prevention focus and promotion focus, when implementing ESM initiatives. This study demonstrates that these regulatory orientations moderate the connection between ESM usage and worker agility, as well as ESM usage and work engagement. Understanding employees’ regulatory focus can help tailor interventions and strategies that align with their motivations and maximize the effectiveness of ESM platforms. Managers can provide targeted training, support, and incentives to leverage ESM’s benefits for different regulatory focus orientations [5].

## Data Availability

The datasets used and/or analyzed during the current study are available from the corresponding author upon reasonable request.
